# Comprehensibility of gender-fair language in German-language video lectures

**DOI:** 10.3389/fpsyg.2024.1496140

**Published:** 2025-01-07

**Authors:** Marcus C. G. Friedrich, Bianca Krenz

**Affiliations:** Institute of Educational Psychology, Technische Universität Braunschweig, Braunschweig, Germany

**Keywords:** gender-fair language, masculine generics, grammatical gender, video lectures, comprehensibility, intelligibility

## Abstract

In many languages, it is common to use masculine-only forms when all genders are meant or gender is irrelevant to the actual statement. This practice is criticized for making women and members of other genders, their achievements and interests, less visible. Gender-fair language is intended to represent all genders equally. Recently introduced forms such as the glottal stop and the gender star are intended to also represent people outside the male–female dichotomy on the linguistic surface. However, it is often argued that gender-fair language would make texts less comprehensible and less aesthetically appealing. The critics’ assumptions were tested in an experiment with 272 participants. Subjects watched a screencast on self-regulated learning and were randomly assigned to either a version using masculine-only forms or a version using the glottal stop and the gender star. Subsequently, participants rated the comprehensibility and aesthetic appeal of the video they had watched. Structural equation models show no statistically significant influence of the use of gender-fair language on the comprehensibility (*β* = −0.13) or the aesthetic appeal (*β* = −0.16) of the videos. The critics’ assumptions are therefore not supported. But further studies are needed, especially regarding the corresponding singular forms and with non-academic participants.

## 1 Introduction

In many languages, it is common to use masculine-only forms when all genders are meant or gender is irrelevant to the actual statement (Stahlberg et al., 2007). This practice is criticized for making women and members of other genders, as well as their achievements and interests, less visible (Saul and Diaz-Leon, 2018). Yet it is often argued that gender-fair language makes texts less comprehensible and less aesthetically appealing (e.g., Payr, 2022). The present paper examines how the comprehensibility and aesthetics of video lectures are influenced by the gender star and the glottal stop as special forms of (spoken) gender-fair language that are intended to make the non-binary genders in particular linguistically visible.

The following section describes how gender is represented in different languages, how comprehension proceeds, and how the representation of gender in language influences mental representations, motivational variables, and behavior. This is followed by a discussion of what comprehensibility is and what effects of gender-fair language on comprehensibility were shown in previous studies. On this basis, hypotheses regarding the influence of gender-fair language on comprehensibility are derived, then tested in an experiment and discussed.

### 1.1 Representation of gender in language

Zacharski and Ferstl (2023) distinguish three sources of information about a person’s gender in German: lexico-semantic, conceptual, and grammatical information (*cf.* Diewald and Steinhauer, 2020). The gender of the person being referred to is part of the meaning of words (lexico-semantic information). The word “aunt,” for example, by definition refers to a female person, while the word “uncle” refers to a male person. Words are also associated with stereotypes (conceptual level), which primarily depend on an individual’s experiences, which are in turn influenced by the individual’s social and cultural environment. Psychology, for example, is typically a female profession and therefore more strongly associated with women, while engineering is typically a male profession and therefore more strongly associated with men (*cf.* Gottfredson, 2005). Finally, grammar can also contain information about the gender of the persons referred to. Gender is represented very differently in the languages of the world, however. Gygax et al. (2019) divide languages into five categories with regard to the representation of gender. Table 1 provides an overview of these categories. In grammatical gender languages such as German, all words are thus associated with a specific gender. For inanimate objects, the gender of the word is assigned arbitrarily, but for animate objects, the grammatical gender is strongly associated with the gender of the persons being referred to (Diewald and Steinhauer, 2020).

**Table 1:** Overview of the representation of gender in the languages according to Gygax et al. (2019).

Category	Examples	Features
Genderless languages	Chinese, Finnish, Turkish	Only a few nouns are associated with a specific gender.
Genderless languages with a few traces of grammatical gender	Basque, Oriya	Most nouns and personal pronouns are not associated with a specific gender, but some nouns, adjectives, and verb forms are marked by suffixes.
Natural gender languages	English	Most personal pronouns are associated with a specific gender, but most nouns are not.
Languages with a combination of grammatical gender and natural gender	Dutch, Norwegian	Personal Pronouns and some nouns are typically associated with a specific gender.
Grammatical gender languages	German, Hebrew, Spanish, Ukrainian	Almost all nouns and personal pronouns are associated with a specific gender.

It is common practice in all languages to use the masculine form to also refer to all genders or when the gender is irrelevant to the actual statement (Stahlberg et al., 2007). This practice is often referred to as “generic masculine,” in contrast to the “specific masculine,” which is used to refer to males only. The generic and the specific forms are formally identical and only differ due to the author’s intention. Whether masculine-only forms are meant generically or specifically must be inferred from the context. This practice has therefore been criticized for setting masculinity as the norm (MAN) and making women and members of other genders, as well as their achievements and interests, less visible (Saul and Diaz-Leon, 2018).

Gender-fair language, on the other hand, aims to represent all genders equally. Sczesny et al. (2016) distinguish two strategies for the implementation of gender-fair language: neutralization and feminization. Neutralization strategies aim to use neutral expressions such as *Lehrkräfte* (“teaching staff”) in which the gender is not explicitly addressed. Feminization strategies, on the other hand, aim to make the feminine form explicitly visible, for example by using pair forms, e.g., *die Lehrerin bzw. Der Lehrer* [“the teacher (female) or the teacher (male)”]. Table 2 provides an overview of common forms of gender-fair language in German (*cf.* Diewald and Steinhauer, 2020).

**Table 2:** Overview of typical forms of gender-fair language in German.

Category	Example	Translation
Neutral forms	*die Lehrkräfte*	the teaching staff
Substantiated participles	*die Lehrenden*	~ those who teach
Pair forms	*die Lehrerinnen und Lehrer*	the teachers (female) and teachers (male)
Slash forms	*die Lehrer/innen*	~ the fe/male teachers
Internal-I forms	*die LehrerInnen*	~ the feMale teachers
Gender gap	*die Lehrer_innen*	~ the fe_male teachers
Gender colon	*die Lehrer:innen*	~ the fe:male teachers
Gender star	die Lehrer*innen	~ the fe*male teachers
Glottal stop	*die Lehrerʔinnen*	~ the feʔmale teachers

The gender gap, the gender colon, and the gender star are written forms of gender-fair language, which are often spoken using the glottal stop, a form of spoken gender-fair language in German.

Neutral forms and substantiated participles constitute neutralization strategies. Slash forms and internal-I forms are considered abbreviated pair forms; all three are considered to be feminization strategies. Recently, these forms have been criticized for not adequately representing genders outside the male–female dichotomy. Therefore, newer forms of gender-fair language have been introduced to also represent these genders on the linguistic surface, e.g., the gender gap, the gender star, and the gender colon for written texts, and the glottal stop for spoken texts. When using the gender star, a special character is placed between the word stem or the masculine form of the word and its feminine ending, e.g., die Lehrer*innen (~“the fe*male teachers”). The gender gap, the gender colon, and the gender star are often translated into spoken language by using the glottal stop, an abrupt and sustained closure of the vocal cords in the larynx, placed between the word stem or the masculine form of the word and the feminine ending (Garellek, 2013; Völkening, 2022); in the international phonetic alphabet, the glottal stop is represented by the symbol “ʔ,” e.g., *die Lehrerʔinnen*, (~“the feʔmale teachers”).

On the basis of established theories of comprehension, one can expect that masculine-only forms for the representation of all genders and gender-fair language produce different effects. The following section therefore describes the construct of comprehension.

### 1.2 Comprehension

“Comprehension” refers to both a product and a process. The product of comprehension is achieved when a person has built up an appropriate mental representation of an object. This product can be used to derive further conclusions, make predictions, and mentally anticipate the behavior of an object (Schnotz and Bannert, 2003). The process of comprehension describes the creation of the product of comprehension. Comprehension is a central cognitive process that influences perception, thinking, problem solving, and behavior (McNamara and Magliano, 2009). The construction-integration model (Kintsch, 1988, 1998) is considered the most complete and best elaborated theory of comprehension (McNamara and Magliano, 2009). The model assumes that the contents of long-term memory are represented in the form of a semantic network in which objects are represented by nodes and the relationships between the objects by links between the nodes. According to the model, texts are processed in cycles. Each cycle in turn consists of a construction phase and an integration phase, whereby the construction phase is further divided into four steps. In the first step, information from the environment, e.g., a text, is read in and translated into propositions. Propositions assign properties to objects or relate objects to each other. In the second step, associations from the long-term memory are activated on the basis of the content that was loaded into the working memory in the first step. In the third step, gaps in the current representation are automatically closed by further associations (if possible). Finally, in the fourth step of the construction phase, weights are assigned to the content currently represented in the working memory. Positive weights between two representations indicate that the contents are consistent according to previous knowledge or were often activated together in the past; negative weights between two representations indicate that the representations are less consistent according to previous knowledge or contradict each other, for example. The more these weights deviate from zero, the more the representations support or suppress each other. In the integration phase, these weights are finally redistributed in order to achieve an unambiguous and coherent representation by suppressing inappropriate representations and reinforcing appropriate ones. These two phases can be supplemented by further, conscious processes, namely inferences, reinstatements, and reorganizations. Inferences refer to the generation of further propositions with the help of prior knowledge. Reinstatements refer to the reintroduction into the working memory of information that has already been processed but not held in the working memory for further processing. Finally, reorganization refers to processes that are necessary to correct misconceptions about the content or the further course of a text (Kintsch, 1988, 1998).

### 1.3 Effects of the representation of gender on mental representations

From the construction-integration model (and other theories of comprehension, *cf.* McNamara and Magliano, 2009) it follows that masculine-only forms and gender-fair forms have different effects. Since masculine-only forms always refer to males and only sometimes to females, one can expect that the associations of masculine-only forms with males are stronger than with females and persons of other genders. Hence, masculine-only forms trigger more associations with male persons in the second step of the construction phase and these associations are given stronger weights than associations with women and persons of other genders in the fourth step of the construction phase. As a result, associations with men are more likely and more strongly present at the end of the integration phase (Friedrich et al., 2022). Although these mental representations can be corrected through conscious processes such as inferences and reorganizations, these additional processes tend to be avoided in the sense of cognitive economy and are only carried out if the construction of a coherent mental representation is otherwise not possible (*cf.* Schnotz, 1994).

On the other hand, one can expect the use of gender-fair language to lead to more appropriate mental representations with regard to gender, as it avoids uncertainty with regard to the gender of the person being referred to, does not make any reference to a specific gender, or explicitly refers to all genders on the linguistic surface.

A large number of studies with very different methodological approaches show that the use of masculine-only forms leads readers and listeners to form more mental representations of males (the so-called “male bias”) while gender-fair forms lead to the avoidance of this male bias and to the formation of more appropriate mental representations regarding the genders (see for example Braun et al., 2005; Esaulova et al., 2017; Gabriel et al., 2008; Gastil, 1990; Gygax and Gabriel, 2008; Gygax et al., 2008; Hamilton, 1988; Heise, 2000; Horvath et al., 2016; Irmen and Kurovskaja, 2009; Irmen and Linner, 2005; Irmen and Roßberg, 2004, 2006; MacKay and Fulkerson, 1979; Miller and James, 2009; Sato et al., 2016; Sato et al., 2016).

Recently there has also been increased research on the effects of newer forms of gender-fair language, which are intended to also represent genders outside the male–female dichotomy. These studies show that newly introduced gender-neutral pronouns such as “hen” in Swedish or “ze” in English lead to more balanced mental representations of the genders than masculine forms or traditional neutral forms (Lindqvist et al., 2019). The gender star and the glottal stop break the male bias, but also lead to the mental overrepresentation of women (Körner et al., 2024; Körner et al., 2022). Yet the gender star leads, as intended, to members of genders outside the male–female dichotomy being more appropriately represented mentally (Zacharski and Ferstl, 2023). Due to the close links between cognition, motivation, and behavior (*cf.* McNamara and Magliano, 2009), in addition to these effects of the linguistic representation of gender on mental representations, one can also expect effects on motivational variables and behavior.

### 1.4 Effects of the representation of gender on motivation and behavior

When masculine-only forms are used, it is always certain that males are meant, but there is some uncertainty as to whether females or members of other genders are also meant. On the basis of the construction-integration model (Kintsch, 1988, 1998; see above), it can be expected that masculine-only forms can be more easily aligned with notions of men than with notions of women and members of other genders, and that this results in male-oriented behavior. And indeed, these assumptions are supported by a large number of studies with very different methodological approaches.

Women show more interest, higher commitment, and higher self-efficacy regarding typically male occupations when the occupations are presented in gender-fair language instead of masculine-only forms (Bem and Bem, 1973; Metaxa-Kakavouli et al., 2018). Girls and boys also show higher self-efficacy regarding typically male occupations if the occupations are presented in gender-fair language instead of masculine-only forms (Vervecken and Hannover, 2015). Studies involving mock job interviews show that women show a greater sense of belonging, more expected identification, and higher motivation when gender-fair language is used in the interview instead of masculine-only forms (Stout and Dasgupta, 2011). In line with the construction-integration model, women are perceived to have a better fit with high-status jobs and a higher likelihood of success in typically male occupations if the occupations are presented in gender-fair language rather than masculine-only forms (Horvath and Sczesny, 2015; Vervecken et al., 2015; Vervecken et al., 2013). A study by Prewitt-Freilino et al. (2012) shows that the more frequently gender is present in a language (*cf.* Table 1), the larger the wage gap between men and women and the smaller the share of men in domestic work. Finally, gender-fair language reduces stereotypical thinking (Kollmayer et al., 2018). Thus there are good reasons to use gender-fair language. Nonetheless, it is often argued that the use of gender-fair language would make texts less comprehensible and less aesthetically appealing (Blaubergs, 1980; Payr, 2022; Vergoossen et al., 2020). This raises the question of what comprehensibility actually is.

### 1.5 Comprehensibility

Comprehensibility can be defined as the ease with which readers can execute the processes needed to comprehend a certain text (Friedrich, 2017; Kintsch and Vipond, 1979). There are two approaches to comprehensibility in the literature. The dominant approach considers comprehensibility as a characteristic of texts. This approach thus equates text comprehensibility with text complexity. The other, newer approach considers comprehensibility as a characteristic of the text-reader interaction. This concept of comprehensibility is therefore also known as “relative comprehensibility” or the “interactionist view of comprehensibility” (Friedrich and Heise, 2022).

On the side of the text, the following features are particularly important with respect to comprehensibility: word length, word frequency, sentence length, the complexity of the syntax, global and local cohesion of the text, and coherence-building aids such as headings and other indications of the structure of the text and the relevance of its content (Friedrich, 2017; McNamara et al., 2014). Word length is a good proxy for word frequency and sentence length is a good proxy for the complexity of the syntax of the sentences (Collins-Thompson, 2014). Short words and short sentences place less of a burden on the working memory than longer words and longer sentences (Hulme et al., 1996; Service, 1998). Words that occur frequently in a language are more familiar to readers or listeners, making it easier for them to process these words and assign meaning to them (Hulme et al., 1995). In order to comprehend a text, the assignment of word meanings and the decoding of syntax are fundamental processes (Hoover and Gough, 1990). Accordingly, word length and sentence length are simple but powerful predictors of actual comprehension (Friedrich and Heise, 2022; Friedrich and Zimmermann, 2023).

However, how easy it is to comprehend a text depends not only on characteristics of the text, but also on characteristics of the readers, which include their prior knowledge, their vocabulary, the size of their working memory, their motivation, and the reading strategies they use (Cromley and Azevedo, 2007; Friedrich, 2017). Texts are therefore not comprehensible by themselves; they are comprehensible to someone. This notion also corresponds to our everyday experience. A text might be incomprehensible to students during the first semester, but comprehensible at the end of their studies. The text itself has not changed but, for example, the students’ knowledge has. Following this interactionist view of comprehensibility, Friedrich (2017) developed a questionnaire based on the reinterpretation of the comprehensibility concept of Kintsch and Vipond (1979) against the background of the construction-integration model (Kintsch, 1988, 1998) and the comprehensibility concepts developed by Langer et al. (1974) and Gagné and Bell (1981). The questionnaire reliably and validly assesses six characteristics of comprehensibility: the ease with which readers can assign meaning to the words of a text (word difficulty), the ease with which readers can decode the syntax of sentences and translate them into propositions (sentence difficulty), the effort that readers have to invest to correct misconceptions about the content or the further course of the text (effort for reorganizations), the ease with which readers can build a mental model of the text content (clarity of representations), how aesthetically appealing readers find the text (aesthetic appeal/variety of language use), and finally, an overall judgment of how easily readers can comprehend the text as a whole (subjective comprehensibility). The characteristic “aesthetic appeal” is derived from concepts of comprehensibility developed by Langer et al. (1974) and Groeben (1972; *cf.* Ballstaedt and Mandl, 1988), who assume that aesthetically appealing texts are more motivating for readers, thus providing more resources for processing the texts, which in turn make the texts easier to comprehend. With regard to the debate regarding the comprehensibility of gender-fair language, the following dimensions are of particular importance: subjective comprehensibility, word difficulty, sentence difficulty, and aesthetic appeal.

### 1.6 Studies of the influence of gender-fair language on comprehensibility

Critics often argue that gender-fair language would make texts less comprehensible and less aesthetically appealing (Blaubergs, 1980; Payr, 2022; Vergoossen et al., 2020). These assumptions are plausible: gender-fair forms are longer overall and occur less frequently within languages than masculine-only forms. The shorter and more familiar words are, the easier it is to assign meaning to them. Gender-fair forms usually require longer sentences and sentences with a more complex syntax. The shorter and syntactically simpler sentences are, the easier it is to decode their syntaxes. The sentence *Die Schüler lesen* (“The pupils read”) contains the masculine-only phrase *die Schüler* (the pupils) and can be rephrased in a gender-fair way as Die Schüler*innen lesen (~ “The fe*male pupils read”), for example. The phrase “die Schüler*innen” is longer and less common than the masculine-only form and also makes the sentence longer. Word length, word frequency, sentence length, and syntactic complexity are important predictors of the comprehensibility of texts (Friedrich and Heise, 2022; Friedrich and Zimmermann, 2023; McNamara et al., 2014). It is therefore reasonable to assume that gender-fair language makes it more difficult to assign meaning to the words, to decode the syntax of sentences, and thus to comprehend the text as a whole. Furthermore, stimuli are evaluated more positively, the easier it is to process them (Reber and Greifeneder, 2017). One can therefore also assume that gender-fair language impairs the aesthetic appeal of texts. A number of studies have tested these assumptions of the critics of gender-fair language (for an overview see Friedrich and Heise, 2019; see Supplementary Table S1). All these studies were conducted in German and most of them used experiments with a between-subjects design, in which the effect of different forms of gender-fair language was compared to the effect of masculine-only forms on comprehensibility and the aesthetic appeal of the texts. Table 3 provides an overview of the effect sizes of these experiments.

**Table 3:** Overview of effect sizes *d* in experiments comparing different forms of gender-fair language with masculine-only forms in German.

Gender-fair form						
Neutral forms	0.22 ^g^	−0.10 ^g^	−0.21 ^j^			
Pair forms	0.19 ^g^	−0.06 ^d^	−0.12 ^b^	−0.13 ^g^	−0.26 ^d^	−0.27 ^a^
Slash forms	−0.59 ^h^	−0.67* ^j^	−0.72* ^h^			
Internal-I forms	−0.08 ^b^	−0.23 ^a^				
Gender star singular	−0.56* ^c^	−1.05* ^f^	−1.16* ^f^			
Gender star plural	0.38 ^c^	−0.04 ^i^	−0.15 ^f^	−0.49* ^f^		
Glottal stop	−0.15 ^e^	−0.37 ^g^	−0.79* ^g^			

Each cell shows the standardized mean difference Cohen’s *d*. In each case, the condition with the respective type of gender-fair language is compared with a condition with masculine-only forms. Each row is sorted by the magnitude of the effect sizes. Positive values indicate that the gender-fair form was more comprehensible than the masculine-only form, while negative values indicate that the gender-fair form was less comprehensible. The superscript letters indicate the source from which the effect size was taken or derived. ^a^ Blake and Klimmt (2010); ^b^ Braun et al. (2007); ^c^ Friedrich et al. (2021); ^d^ Friedrich and Heise (2019); ^e^ Friedrich et al. (2022); ^f^ Friedrich et al. (2024); ^g^ Jöckel et al., 2021; ^h^ Klimmt et al. (2008); ^i^ Pabst and Kollmayer (2023); ^j^ Pöschko and Prieler (2018); *p < 0.05.

The experiments by Gygax and Gesto (2007), Rothmund and Christmann (2002), as well as Steiger-Loerbroks and von Stockhausen (2014) showed no impairment of comprehensibility through the use of gender-fair language, but they did not provide complete enough information to calculate the corresponding effect sizes. The study by Frank-Cyrus and Dietrich (1997) showed statistically significant small to medium impairments of comprehensibility due to the use of gender-fair language, but since the study was conducted using a within-subjects design, it is reasonable to assume that the answers were influenced by implicit theories of the participants (Friedrich and Heise, 2019; *cf.* Podsakoff et al., 2003).

Only three of the experiments also investigated whether the gender of the reader has an influence on the comprehensibility of masculine-only forms and gender-fair language. Two of these experiments found no effects (Blake and Klimmt, 2010; Rothmund and Christmann, 2002), while one experiment indicated that texts with masculine-only forms are more comprehensible for men than for women (Braun et al., 2007).

A few experiments also investigated the influence of gender-fair language on aesthetic appeal. For example, experiment 2 by Friedrich et al. (2021) and the experiment by Rothmund and Christmann (2002; with *p* < 0.10, however) showed a statistically significant impairment of the aesthetic appeal, while the experiments by Blake and Klimmt (2010), Friedrich and Heise (2019), Friedrich et al. (2022), Pabst and Kollmayer (2023), as well as experiment 1 by Friedrich et al. (2021) showed no statistically significant effect on aesthetic appeal.

Most studies used single-items or questionnaires to assess comprehensibility, but two experiments (also) assessed eye movements (Gygax and Gesto, 2007; Steiger-Loerbroks and von Stockhausen, 2014). In these studies, the readers took longer to process the first passages in gender-fair language, but quickly became accustomed to these forms and then took just as long to process the passages in gender-fair language as they needed to process passages with masculine-only forms.

The available studies have a number of shortcomings, however (*cf.* Friedrich and Heise, 2019). Many studies did not use validated instruments to assess comprehensibility (Blake and Klimmt, 2010; Braun et al., 2007; Frank-Cyrus and Dietrich, 1997; Jöckel et al., 2021; Klimmt et al., 2008; Pöschko and Prieler, 2018; Rothmund and Christmann, 2002) and used samples that were too small to detect small effects (Braun et al., 2007; Friedrich et al., 2022; Gygax and Gesto, 2007; experiment 2 of Jöckel et al., 2021; Klimmt et al., 2008; Pöschko and Prieler, 2018; Steiger-Loerbroks and von Stockhausen, 2014).

Finally, all of the studies cited also used significance tests from the family of regression analysis or analysis of (co-)variance to test the hypotheses. These methods have the advantage that they are familiar to most researchers and are therefore easy to interpret. However, these methods make a number of assumptions that are usually not tested or even violated, in particular the assumption that the dependent variable was measured without measurement error. Breitsohl (2019) therefore recommends that experiments such as these should be analyzed with the help of structural equation models. These take the measurement errors into account, thus allowing the calculation of effect sizes that are adjusted for the measurement errors and exhibit greater statistical power. In addition, most studies examined the comprehensibility of gender-fair language in written texts, but only three experiments examined the effects of spoken gender-fair language (Friedrich et al., 2022; Jöckel et al., 2021). There is thus a lack of studies of the influence of gender-fair language in videos, i.e., experiments in which spoken and written texts are presented.

### 1.7 The processing of spoken texts and videos

As Friedrich et al. (2022) point out, the comprehension of written and spoken texts is similar, but not identical. The construction-integration model (Kintsch, 1988, 1998) assumes that the processing of spoken and written texts is essentially the same. As Niegemann et al. (2008) argue, however, in the case of written texts it is easier to adjust the speed of processing, pause or jump back in the text than in the case of spoken texts or videos. Comprehending spoken texts or videos also typically requires constant attention (Niegemann et al., 2008). It is therefore questionable whether the results regarding the comprehensibility of gender-fair language can be generalized from written texts to spoken texts or videos (Friedrich et al., 2022). Jöckel et al. (2021) examined the comprehensibility of the glottal stop in the introduction of spoken news reports in two experiments. They found an impairment of comprehensibility due to the glottal stop in their experiment with adults with *d* = −0.79 (*p* < 0.05), but not in their experiment with children and adolescents with *d* = −0.37 (n.s.). The experiment by Friedrich et al. (2022) also showed no impairment of the comprehensibility (partial η^2^ < 0.01, n.s.) or the aesthetic appeal (partial η^2^ = 0.02, n.s.) of spoken texts, but with *N* = 97 its sample size was too small to detect such small effects. The hypotheses are therefore tested again with a larger sample and an improved statistical analysis.

### 1.8 Hypotheses

The present experiment tests the critics’ assumptions (as already tested by Friedrich et al., 2022) that gender-fair language impairs comprehensibility and aesthetic appeal (see section 1.6). Accordingly, the following hypotheses are tested: Videos with masculine-only forms are more comprehensible than videos in gender-fair language (*H*_comprehensibility_). Viewers can assign meaning to the words of a video with masculine-only forms more easily than they can in videos with gender-fair language (*H*_word_difficulty_). Viewers can decode the syntax of sentences in videos with masculine-only forms more easily than they can in videos with gender-fair language (*H*_sentence_difficulty_). Videos with masculine-only forms are more aesthetically appealing than videos with gender-fair language (*H*_aesthetic_appeal_). In addition to these hypotheses, the experiment also tests the possible effects of interactions of the language form (masculine-only forms vs. gender-fair language) with the gender of the test subjects (male vs. non-male) on the dependent variables.

Women and members of other genders have more positive attitudes than men toward gender-fair language (Frank-Cyrus and Dietrich, 1997), and one earlier study showed an interaction effect with the gender of the reader (Braun et al., 2007), but others did not (Blake and Klimmt, 2010; Gygax and Gesto, 2007; Rothmund and Christmann, 2002), possibly due to a low statistical power. Therefore, the experiment also examines whether there is an interaction between the linguistic representation of gender in videos (masculine-only forms vs. gender-fair forms) and the viewers’ gender.

Prior knowledge is a substantial predictor of comprehensibility and actual comprehension (Friedrich and Heise, 2022; Cromley and Azevedo, 2007). Therefore, self-assessed prior knowledge about the topic of the videos was recorded as a control variable. Attitudes toward gender-fair language are an important predictor of the use of gender-fair language (Sczesny et al., 2015) and could therefore also influence familiarity with gender-fair language and hence its comprehensibility (Pabst and Kollmayer, 2023). Attitudes toward gender-fair language and past behavior regarding gender-fair language were thus included as control variables.

## 2 Method

The experiment was conducted in German and was approved by the ethics committee of Faculty 2 of the TU Braunschweig (identification number BA_2022–16). We report how we determined our sample size, all data exclusions, all manipulations, and all measures. The materials, scale manuals, data sets, and syntaxes used in this experiment are available at https://doi.org/10.17605/OSF.IO/XPR4E

### 2.1 Participants

The study was advertised via various e-mail distribution lists at universities and universities of applied sciences in Lower Saxony as well as on social media. Psychology students were eligible to receive credits for participating in the study. All others were eligible to take part in a draw for five vouchers worth 20 euros each. Three hundred and twenty-six people took part in the study. Forty-three people were excluded because they watched the video for less than 8 min; 8 persons because they did not indicate for how long they had watched the video; 2 persons because they indicated in the open answers of the questionnaire that they were doing something else on the side; and 1 person because he did not answer any of the scales completely. The final sample consisted of 272 persons (161 females, 111 males, 0 diverse); 173 were between 18 and 25 years old, 62 between 26 and 35, 15 between 36 and 45, and 22 were 45 years old or older. One person had no school-leaving certificate, 1 had a lower secondary school leaving certificate, 7 had an intermediate secondary school leaving certificate, 195 had a (specialized) high school diploma, 67 had a bachelor’s or master’s degree, and 1 person did not specify their highest level of education. The specialized high school diploma (*Fachabitur*) is an educational qualification that allows students to study at universities of applied sciences and certain study programs at universities in Germany.

### 2.2 Materials

The videos used showed screencasts about self-regulated learning, i.e., the videos showed screen recordings of a PowerPoint presentation in which the female speaker was not visible. The presentation was spoken and recorded individually in two versions. One version used masculine-only forms, i.e., the slides showed personal identifiers in masculine-only forms in 24 instances and the spoken text contained a total of 36 personal identifiers in masculine-only forms, namely *die Studenten* (“the students”; 15 written and 23 spoken instances), *die Dozenten* (“the lecturers”; 3 and 5), *der Lernende* (“the learner”; 3 and 4), *die (Lern-)Forscher* (“the (learning) researchers”; 2 and 3), and *die Experten* (“the experts”; 1 and 1). This version of the video was 13:46 min long. In the following text, this version is referred to as the video with masculine-only forms (MOF). The other version of the video used gender-fair forms, i.e., the masculine-only forms were replaced by forms with the gender star on the slides (e. g. die Student*innen, ~“the fe*male students”) and in the spoken text masculine-only forms were replaced by forms with the glottal stop, namely *die Studentʔinnen* (~“the feʔmale students”), *die Dozentʔinnen* (~“the feʔmale lecturers”), *die Lernenden* (~“those who learn”), *die (Lern-)Forscherʔinnen* (~“the feʔmale (learning) researchers”), and *die Expertʔinnen* (~“the feʔmale experts”). This version is referred to below as the video with gender-fair forms (GFF). This version was 11:46 min long. The spoken texts were 1,571 words long in both videos.

### 2.3 Instruments

The comprehensibility of the videos was assessed using scales from the comprehensibility questionnaire by Friedrich (2017), as adapted by Friedrich et al. (2022). The comprehensibility of the videos was assessed using the adapted scale of *subjective comprehensibility* (three items; sample item: *Ich fand das Video verständlich*. “I thought the video was comprehensible.”). The ease with which viewers could assign meaning to the words in the videos was measured using the adapted scale *word difficulty* (three items; sample item: *Bei manchen Wörtern war ich mir nicht sicher, was sie bedeuten*. “For some words, I was not sure what they meant.”). The ease with which viewers could decode the syntax of the sentences was measured using the adapted scale *sentence difficulty* (three items; sample item: *Die Sätze waren kompliziert gebaut*. “The sentences had a complicated structure.”). Finally, the aesthetic appeal of the videos was assessed using the adapted scale var*iety of language use* (three items; sample item: *Ich fand die Sprache lebhaft*. “I thought the language was lively.”).

Self-ascribed prior knowledge was assessed as a control variable using a translation of the scale *background knowledge* developed by Harackiewicz et al. (2008) (three items; sample item: *Ich hatte bereits ein gewisses Vorwissen zum selbstregulierten Lernen (z. B. habe ich dazu etwas in einer Lehrveranstaltung gelernt oder mich selbst informiert)*. “I already had some prior knowledge of self-regulated learning (e.g., I learned something about it in a course or I found out the information myself)”).

Attitudes toward gender-fair language were measured as a control variable using the scale *positive attitudes* developed by Sczesny et al. (2015; five items; sample item: *Geschlechtergerechte Sprache zu verwenden, ist mir persönlich wichtig*. “It is important for me personally to use gender-fair language.”).

Past behavior regarding gender-fair language was assessed as a control variable using an adaptation of the scale *past behavior* developed by Sczesny et al. (2015; four items; sample item: *Geschlechtergerechte Sprache habe ich in den letzten Monaten im privaten Bereich in schriftlicher Form verwendet*. “I have used gender-fair language in writing in my private life over the last few months.”).

Participants were asked whether they had watched the video in full or how many minutes of the video they had watched. Furthermore, they were asked to make guesses about the hypothesis that was being tested in the study. At the end, the participants’ gender, age, and highest level of education were collected. Finally, subjects were given the opportunity to make comments on the videos or the study at the end of the survey.

### 2.4 Procedure

The study was conducted as an experiment using a between-subjects design with the factors linguistic representation of gender in the video (masculine-only forms, MOF, vs. gender-fair forms, GFF) and gender of the test subject (male vs. non-male). The experiment was conducted online on the platform Unipark.com.

The procedure corresponded to that of Friedrich et al. (2022). The subjects were first welcomed and informed about data protection as well as the voluntary nature of their participation. After giving their informed consent, they randomly saw one of the two versions of the video, either the version using masculine-only forms (MOF) or the version using gender-fair language (GFF). Afterwards, the participants were asked whether they had watched the full video or how many minutes of the video they had watched, before completing the comprehensibility questionnaire. The subjects were then asked to guess the hypothesis that the study was testing before being informed that they would not be able to return to previous pages from the following page onwards. The participants then completed the scale regarding attitudes toward gender-fair language and answered questions about their gender, age, and highest level of education. Finally, they had the opportunity to make comments on the video and the study.

### 2.5 Statistical analysis

**Figure 1:**
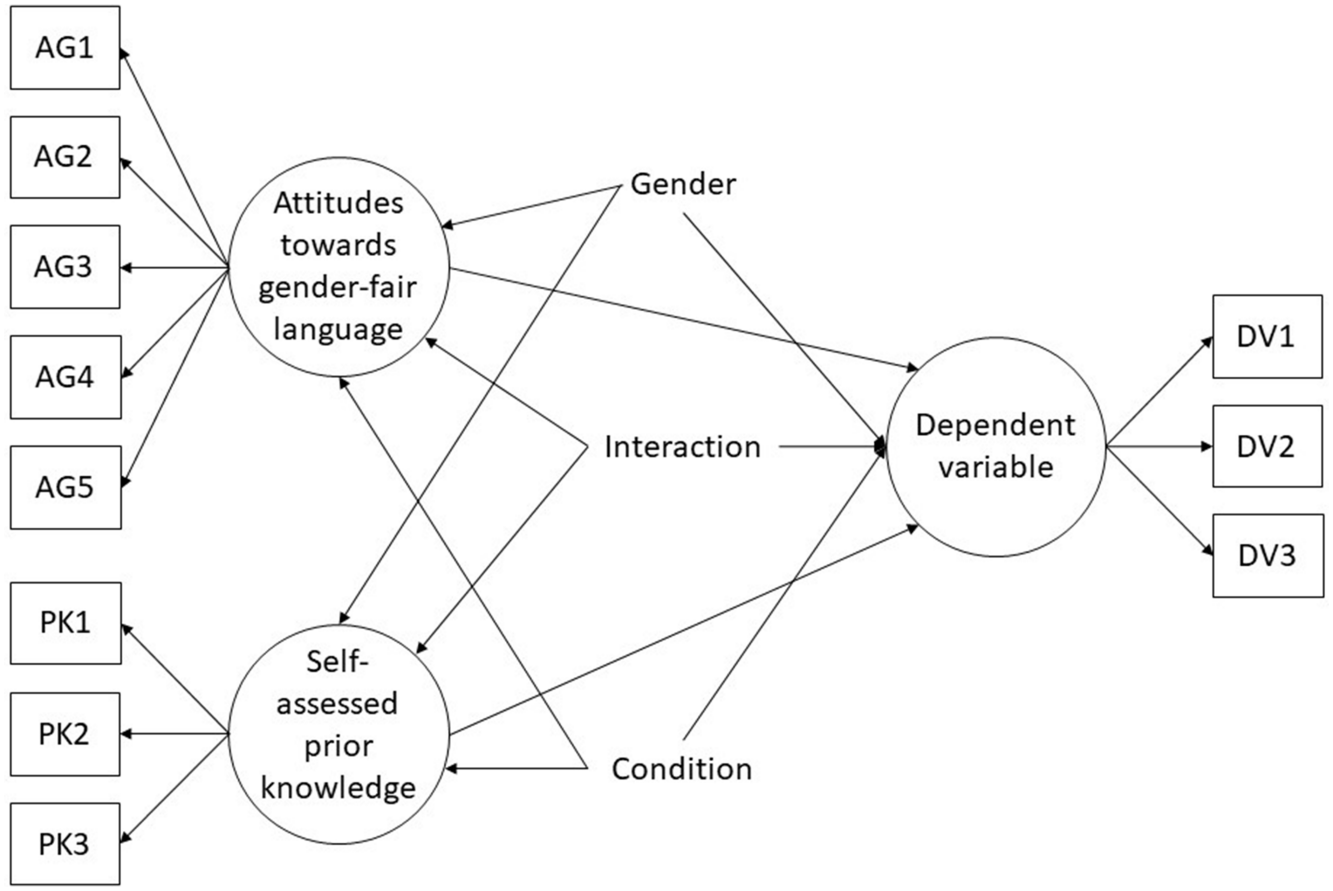
Visualization of the structural equation models tested. In the complete models, the paths from the experimental condition and the interaction to the dependent variable are estimated freely. In the more restrictive models, the paths from the experimental condition and the interaction to the dependent variable are each fixed at zero.

The data was analyzed using structural equation models (SEMs), more specifically using the *multiple indicators multiple causes model* (MIMIC) laid out by Breitsohl (2019). Analyzing the data of the experiment using SEMs instead of ANOVAs has several advantages, in particular the explicit accounting of measurement errors of the dependent variables and the higher statistical power of the significance tests. Two structural equation models are calculated for each dependent variable, namely (1) a full model in which the influence of the experimental condition (masculine-only forms vs. gender-fair language) and its interaction with the participants gender are freely estimated and (2) a more restrictive model in which the corresponding path coefficients are fixed to zero. Within the full model, the dependent variable is predicted by the experimental condition, the participants’ gender, and the interaction of the experimental condition with the gender as well as the control variables (see Figure 1). The second, more restrictive model represents the null hypothesis.

The hypotheses are then tested in three ways: (1) by a significance test of the path coefficients from the experimental condition as well as its’ interaction with the participants gender on the dependent variable; (2) by comparing the fit indices of the two models; and (3) by comparing the corrected Akaike information criteria (AICcs) of the two models.

Ad 1: If gender-fair language has a significant effect on the dependent variables, the path coefficients of the experimental conditions or its interaction with gender within the model should significantly deviate from zero.

Ad 2: If gender-fair language influences the dependent variable, the more restrictive model should fit the data significantly less well than the full model. The corresponding hypothesis is tested with a χ^2^-test here.

Ad 3: The results of the significance tests of the path coefficients and the χ^2^ tests also depend on the sample size. The two models are therefore also compared with each other using their AICcs. The AICcs describe the distance between a model and a model that fully describes reality. Since reality is unknown, the AICc values cannot be interpreted by themselves, but the distances of the AICcs between different models can. The distance between the models is specified using ΔAICc. Models with a ΔAICc between 0 and 2 can be considered equally well supported, models with a distance of ΔAICc between 4 and 7 still have a certain plausibility compared to each other, and models with a distance of ΔAICc greater than 10 are considered implausible (for a detailed description see Burnham and Anderson, 2002).

## 3 Results

Table 4 shows the means, standard deviations, and internal consistencies for the scales. With internal consistencies between McDonald’s *Ω* = 0.79 and 0.91, the scales proved to be satisfactory to excellent. However, the measurement model for the past behavior did not show sufficient model fit and was therefore omitted for the following analyses.

**Table 4:** Means, standard deviations and internal consistencies of the measured variables.

Scale	*M* (*SD*)	Ω
Subjective comprehensibility	4.02 (0.80)	0.79
Word difficulty	1.74 (0.83)	0.85
Sentence difficulty	2.06 (0.89)	0.84
Aesthetic appeal	2.78 (1.01)	0.87
Self-assessed prior knowledge	3.15 (1.27)	0.88
Attitudes toward gender-fair language	2.61 (1.14)	0.91
Past behavior regarding gender-fair language	2.51 (1.28)	0.88

Scale range: 1 = *stimmt nicht* (“I disagree”) to 5 = *stimmt genau* (“I agree”).

Table 5 shows the intercorrelations of the scales.

**Table 5:** Intercorrelations of the variables.

Scale	SC	WD	SD	AA	PK	AG
Subjective comprehensibility (SC)	–					
Word difficulty (WD)	−0.37***	–				
Sentence difficulty (SD)	−0.64***	0.34***	–			
Aesthetic appeal (AA)	0.46***	−0.03	−0.39***	–		
Self-assessed prior knowledge (PK)	0.15*	−0.25***	−0.11*	−0.05	–	
Attitudes toward gender-fair language (AG)	−0.02	−0.04	0.11*	−0.06	0.10*	
Past behavior regarding gender-fair language	0.13*	−0.10	0.05	0.03	0.18**	0.62***

*p < 0.05, **p < 0.01, ***p < 0.001.

The differences between the two experimental conditions correspond to a small but statistically non-significant effect with respect to gender (MOF: *n_female_* = 68, *n_male_* = 60, GFF: *n_female_* = 93, *n_male_* = 51, *Φ* = 0.12, *df* = 1, χ^2^ = 3.22, *p* = 0.07) and self-assessed prior knowledge (*d* = −0.20, *t_emp_* = 1.65, *df* = 267, *p* = 0.10), and to non-significant null effects with respect to age (Somer’s *d* = 0.01, χ^2^ = 0.01, *df* = 1, *p* = 0.91), level of education (Somer’s *d* = 0.05, χ^2^ = 0.99, *df* = 1, *p* = 0.32), attitude toward gender-fair language (*d* = −0.09, *t_emp_* = −0.72, *df* = 264, *p* = 0.48), and past behavior regarding gender-fair language (*d* = 0.15, *t_emp_* = 1.23, *df* = 269, *p* = 0.22). The randomization was thus considered successful. Table 6 shows the means and standard deviations by the experimental condition and the gender of the participants.

**Table 6:** Means and standard deviations of the variables by experimental condition and participants’ gender.

	Masculine-only forms		Gender-fair forms	
	Females (*n* = 68)	Males (*n* = 60)	Females (*n* = 92)	Males (*n* = 50)
Variable	*M* (*SD*)	*M* (*SD*)	*M* (*SD*)	*M* (*SD*)
Subjective comprehensibility	4.20 (0.76)	3.94 (0.75)	4.06 (0.81)	3.81 (0.92)
Word difficulty	1.79 (0.87)	1.73 (0.73)	1.68 (0.86)	1.80 (0.82)
Sentence difficulty	1.99 (0.88)	2.02 (0.75)	2.07 (0.89)	2.19 (1.07)
Aesthetic appeal	2.87 (0.97)	2.53 (0.91)	2.94 (1.03)	2.65 (1.09)
Self-assessed prior knowledge	3.05 (1.30)	2.95 (1.27)	3.46 (1.23)	2.94 (1.21)
Attitudes toward gender-fair language	2.84 (1.07)	2.24 (0.99)	2.89 (1.25)	2.24 (1.01)
Past behavior regarding gender-fair language	2.76 (1.22)	2.45 (1.23)	2.73 (1.35)	1.85 (1.09)

Scale range: 1 = *stimmt nicht* (“I disagree”) to 5 = *stimmt genau* (“I agree”).

### 3.1 Structural equation models

Table 7 shows the correlation and regression parameters of the different models. Table 8 shows the fit indices of the complete models and the models in which the path from the experimental condition to the dependent variable is fixed at zero in each case. The fit indices indicate a good model fit throughout.

**Table 7:** Standardized regression coefficients of the paths related to the dependent variables in the corresponding structural equation models.

Model	Dependent variable	*β*_prior knowledge, DV_	*β*_attitudes, DV_	*β*_gender, DV_	*β*_condition, DV_	*β*_interaction, DV_
SC	Subjective comprehensibility	0.15*	−0.06	−0.32*	0.15	−0.02
SC*0	Subjective comprehensibility	0.14*	−0.06	−0.30*	–	–
WD	Word difficulty	−0.28***	−0.03	−0.04	−0.02	−0.04
WD*0	Word difficulty	−0.28***	−0.03	−0.04	–	–
SD	Sentence difficulty	−0.14*	0.14*	0.13	−0.17	−0.06
SD*0	Sentence difficulty	−0.14*	0.14*	0.11	–	–
AA	Aesthetic appeal	−0.10	−0.12	−0.39***	−0.16	−0.01
AA*0	Aesthetic appeal	−0.09	−0.12	−0.41***	–	–

The models SC, WD, SD, and AA are the full respective models; the models SC*0, WD*0, SD*0, and AA*0 are models in which the paths from the experimental condition and its interaction with the participants’ gender to the dependent variable are fixed at zero. “prior knowledge”: self-assessed prior knowledge; “attitudes”: attitudes toward gender-fair language; “DV”: dependent variable. “–” Indicates that this path was fixed at zero in this model.

**Table 8:** Fit indices of the structural equation models.

Model	χ^2^	*df*	χ^2^/*df*	*p*	CFI	TLI	RMSEA	95% CI RMSEA	SRMR	AIC
SC	107.33	68	1.58	0.002	0.98	0.97	0.05	[0.03–0.06]	0.04	9238.14
SC*0	108.60	70	1.55	0.002	0.98	0.97	0.05	[0.03–0.06]	0.04	9235.41
WD	127.81	68	1.88	<0.001	0.97	0.96	0.06	[0.04–0.07]	0.04	9068.49
WD*0	127.89	70	1.83	<0.001	0.97	0.96	0.06	[0.04–0.07]	0.04	9064.57
SD	104.07	68	1.53	0.003	0.98	0.97	0.04	[0.03–0.06]	0.04	9265.17
SD*0	105.80	70	1.51	0.004	0.98	0.97	0.04	[0.03–0.06]	0.04	9262.90
AA	124.32	68	1.83	<0.001	0.97	0.96	0.06	[0.04–0.07]	0.04	9348.95
AA*0	125.81	70	1.80	<0.001	0.97	0.96	0.05	[0.04–0.07]	0.04	9346.44

Lines SC, WD, SD and AA show the values for the full models; lines SC*0, WD*0, SD*0, and AA*0 show the models in which the path from the experimental condition and its interaction with gender to the dependent variable is fixed at zero. Line “SC” shows the fit-indices for the model with subjective comprehensibility as the dependent variable, line “WD” with word difficulty, “SD” with sentence difficulty, and “AA” with aesthetic appeal as the dependent variable.

**Figure 2:**
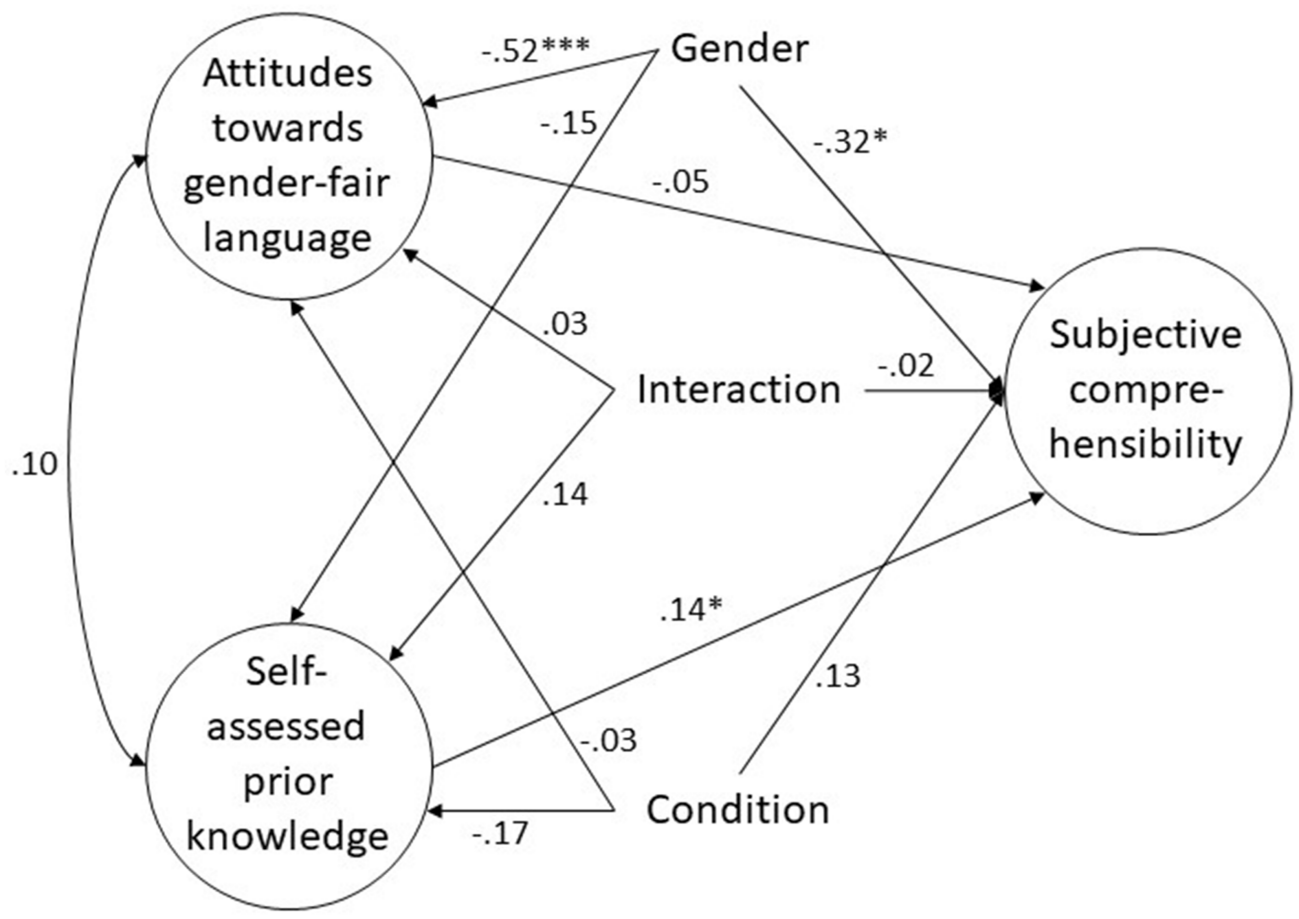
Visualization of the complete structural equation model with subjective comprehensibility as the dependent variable and freely estimated parameters from the independent variable and the covariates on the dependent variable. *p < 0.05, **p < 0.01, ***p < 0.001.

Figure 2 shows the complete model with subjective comprehensibility as the dependent variable as an example for the models. The effect of the experimental condition on subjective comprehensibility corresponds to a statistically non-significant small effect, with *r* = −0.15, *p* = 0.29; the effect of the interaction of the experimental condition and gender on comprehensibility also corresponds to a statistically non-significant null effect, with *r* = −0.02, *p* = 0.86. The χ^2^ test comparing the full model with the more restricted model in which the paths from the experimental condition and its interaction with gender to subjective comprehensibility are fixed at zero is also not statistically significant, with χ^2^ = 1.27, *df* = 2, *p* = 0.53. Finally, a comparison of the AICs shows that the full model is not considerably better than the more restricted model, with ΔAIC = 2.73 (*cf.* Burnham and Anderson, 2002). The hypothesis *H*_comprehensibility_ is therefore rejected.

In the full model the effect of the experimental condition on word difficulty corresponds to a statistically non-significant null effect, with *r* = −0.00, *p* = 0.99; the effect of the interaction on word difficulty also corresponds to a statistically non-significant null effect, with *r* = −0.03, *p* = 0.71. The χ^2^ test comparing the full model with the more restricted model is also not statistically significant, with χ^2^ = 0.08, *df* = 2, *p* = 0.96. Finally, a comparison of the AICs shows that the full model is not considerably better than the more restricted model, with ΔAIC = 3.92. The hypothesis *H*_word_difficulty_ is therefore rejected.

In the full model the effect of the experimental condition on sentence difficulty corresponds to a small but statistically non-significant effect, with *r* = −0.17, *p* = 0.20; the interaction effect also corresponds to a statistically non-significant null effect, with *r* = −0.06, *p* = 0.65. The χ^2^ test comparing the full model with the more restricted model is also not statistically significant, with χ^2^ = 1.73, *df* = 2, *p* = 0.42. Finally, a comparison of the AICs shows that the full model is not considerably better than the more restricted model, with ΔAIC = 2.27. The hypothesis *H*_sentence_difficulty_ is therefore rejected.

In the full model the effect of the experimental condition on aesthetic appeal corresponds to a small but statistically non-significant effect, with *r* = −0.16, *p* = 0.23; the interaction effect also corresponds to a statistically non-significant null effect, with *r* = −0.01, *p* = 0.96. The χ^2^ test comparing the full model with the more restricted model is also not statistically significant, with χ^2^ = 1.49, *df* = 2, *p* = 0.47. Finally, a comparison of the AICs shows that the full model is not considerably better than the more restricted model, with ΔAIC = 2.51. The hypothesis *H*_aesthetic_appeal_ is therefore rejected.

### 3.2 Further observations

If the hypotheses are tested using simple 2 × 2-ANOVAs instead of the structural equation models, the results remain the same (see Supplementary Table S2).

At the end of the study, 64 test subjects took the opportunity to write a comment about the video or the study and commented, for example, on the sound quality of the video or the relevance of the topic to their academic studies. Three participants in the gender-fair language condition group stated that they found the use of gender-fair language annoying. On the other hand, three participants in the masculine-only condition group stated that they would have preferred the use of gender-fair language. These are only a few comments on this topic overall, but they nevertheless show that the subject matters to people (for a complete list of all comments and their translations, see Supplementary Table S3).

## 4 Discussion

This study tested the assumption that gender-fair language reduces the comprehensibility and aesthetic appeal of instructional videos. For this purpose, an experiment with 272 participants was conducted in a between-subjects design and analyzed using structural equation models. Contrary to the critics’ assumptions, the results show no statistically significant impairment of the comprehensibility or aesthetic appeal of the instructional videos due to the use of the gender star and the glottal stop.

The results are thus consistent with the findings of Friedrich et al. (2022), who also found weak but statistically non-significant effects of gender-fair language in instructional videos on comprehensibility and aesthetic appeal. The influence of the glottal stop and the gender star in videos thus appears to be weaker than that of other strategies for using gender-fair language (*cf.* Table 3). The effect in the present experiment and the experiment by Friedrich et al. (2022) is considerably smaller than in the experiments by Jöckel et al. (2021), however. This can possibly be explained by the length of the examined texts or the proportion of manipulated text passages. In the experiments by Gygax and Gesto (2007) and Steiger-Loerbroks and von Stockhausen (2014), the test subjects took longer to process the first passages in gender-fair language, but then quickly became accustomed to it and processed the gender-fair forms just as quickly as masculine-only forms. The material in the experiments by Jöckel et al. (2021) was very short and contained a large number of text passages in which the glottal stop was used in relation to the length of the text (5.88% compared to 2.29% in the present experiment). It is reasonable to assume that the different effects were caused by the different lengths of the material or the different proportion of text passages in gender-fair language. A study of DPA reports (*Deutsche Presse-Agentur*; German Press Agency) and texts in the magazine *Brigitte* indicates that around 1% of all words in texts need to be changed in order to write in a gender-fair way (Müller-Spitzer et al., 2024). In the studies of the influence of gender-fair language on comprehensibility and aesthetic appeal that provide the necessary information, between 2 and 11% of the text required corresponding changes (*Md* = 4.33%). All of the available studies therefore probably overestimate the effect of the use gender-fair language for most texts. The assumption that the number or proportion of text passages in gender-fair language has an influence on the comprehensibility of the texts needs to be explicitly tested in further experiments.

Furthermore, Friedrich et al. (2022) only manipulated the audio track of the videos with respect to the use of gender-fair language. The present study also manipulated the visual track in this regard. The results of both studies are very similar. Nevertheless, it would be interesting to test the effects of gender-fair language in the audio track, the video track and their combination in detail. In addition, it is desirable to examine whether the results can also be generalized to auditory media such as podcasts and radio programs.

The present experiment is subject to a number of limitations, however. Since the video using gender-fair language was about 2 min shorter than the video using masculine-only forms, it is conceivable that the length of the videos had a confounding influence on the evaluation of the videos. The video with masculine-only forms was longer, i.e., 1,571 words in 13:46 min or 114 words per minute, while the video in gender-fair language had 1,571 words in 11:46 min or 133 words per minute. The most efficient listening rate is around 270 words per minute and spoken texts are less comprehensible when they comprise more words per minute (Kuperman et al., 2021). Both texts were thus below the critical range within which comprehension becomes difficult. Since the video in gender-fair language contained more words per minute than the video with only masculine forms, one would have expected the video in gender-fair language to be less comprehensible, given this background as well. Yet, the results should be replicated in experiments where the length of the videos differs less. Furthermore, as both videos were recorded by the same speaker, it cannot be ruled out that the speaker unconsciously spoke differently in the two recordings. Friedrich et al. (2022) therefore created an audio track with masculine-only forms and produced the version with the glottal stop by inserting the syllable “-innen” into the audio track after the masculine-only forms at the respective positions. Only through the manipulation chosen in the present experiment, however, does the manipulation constitute a genuine glottal stop. Future studies could instead use a computer voice to ensure that the audio track contains a glottal stop, but is otherwise identical.

Participants were asked to watch the video and were afterwards asked how long they had watched the video. This presumably corresponds to how educational videos are typically watched. However, it is possible that the subjects watched the video without sound, just listened to it or did something else on the side. In future studies, the test subjects should therefore also be asked whether they read the texts on the screen and whether they listened attentively. In addition, it would be interesting to ask the participants, which forms of gender representation, were used in the video in order to check to what extent they noticed the use of gender-fair language.

The present experiment measured comprehensibility using questionnaires, partly in order to investigate such a large sample economically. Nevertheless, it is desirable to replicate the results using other measurement methods, in particular non-reactive ones such as brain waves and eye movements (*cf.* Podsakoff et al., 2003).

The present experiment primarily examined plural forms, as did most experiments regarding this topic (Rothmund and Christmann, 2002; Braun et al., 2007; Klimmt et al., 2008; Blake and Klimmt, 2010; Gygax and Gesto, 2007; Steiger-Loerbroks and von Stockhausen, 2014; Pöschko and Prieler, 2018). Only the experiments by Friedrich and Heise (2019), Friedrich et al. (2024), and the second experiment by Friedrich et al. (2021) (also) examined singular forms. The singular forms are more complicated in German, since the article in the plural is always *die* (“the (plural)”), but can be *der* (“the (masculine)”), *die* (“the (feminine)”) or *das* (“the (neutral)”) in the singular (Diewald and Steinhauer, 2020). This makes less frequent constructions necessary, e.g., with several articles, e.g., die*der Lehrer*in (~“the (masculine)*the (feminine) teacher”), or – more simply – d. Spieler*in ~ “t. fe*male player.” It is therefore desirable to conduct more experiments in which the singular forms of gender-fair language are examined. Furthermore, additional studies of the comprehensibility of other forms of gender-fair language are desirable, notably regarding neutral forms and substantiated participles (*cf.* Table 3).

Overall, the sample was highly educated and not representative of the German population. It can therefore be assumed that the sample has, for example, greater linguistic skills than samples with a lower level of education and that the sample is more familiar with forms of gender-fair language. It is therefore desirable to replicate the study with other samples, especially with non-academics, schoolchildren, and individuals learning German as a foreign language.

There is still a lack of studies of the mental representations evoked by the glottal stop. Previous studies suggest that the glottal stop causes a female bias (Körner et al., 2024). Yet it is unclear whether it actually leads to the mental inclusion of members of non-binary genders as intended.

Previous studies indicate that gender-fair language is processed differently by women and men. However, so far there are no studies on whether the use of gender-fair language by women, men und non-binary persons is perceived differently by others. It would therefore be desirable for future studies to investigate possible interaction effects of the use of gender-fair language with the gender of the speaker.

Furthermore, the question arises as to how well the results can be generalized to other languages, especially to other grammatical gender languages such as Spanish or French. In Spanish, for example, you can write “alumn@s” or “alumnos/as” in order to write in a gender-fair way. These forms cannot be translated into spoken language as easily as the gender star (e.g., “Student*in”) with the glottal stop (e.g., “Student*in”) in German. However, except for the study by Gygax and Gesto (2007) in French, all studies on the comprehensibility of gender-fair language seem to have been carried out in German.

The main advantage of the present study was its relatively large sample size. With a sample size of *N* = 272, effects as small as *f* = 0.17 could be detected with an *α* = 0.05 and a power of 1 – *β* = 0.80. To detect even smaller effects of size *f* = 0.10 at an α = 0.05 with a power of 1 – *β* =0.80, a sample of size *N* ≥ 620 would be necessary. It would therefore be desirable to collect such large samples in future studies. At the same time, it is unclear what impairment of comprehensibility would be acceptable for a more appropriate representation of gender in language. This question, lies beyond the scope of the present study, though.

If the results of this experiment are supported in further studies, it speaks in favor of using the glottal stop if one wants to break the male bias and make women and presumably members of other genders more visible. At least in their plural forms, the glottal stop and the gender star do not appear to impair the comprehensibility and aesthetic appeal of videos.

## Data Availability

The datasets presented in this study can be found in online repositories. The names of the repository/repositories and accession number(s) can be found at: https://doi.org/10.17605/OSF.IO/XPR4E.
