# Supplementary material for: Comprehensibility of gender-fair language in German-language video lectures
Source: Front Psychol. 2025 Jan 7;15:1496140. doi: 10.3389/fpsyg.2024.1496140 (PMC11745881; doi:10.3389/fpsyg.2024.1496140)
Supplement: Supplementary file 1 [file Table_1.pdf]

Supplementary Material

1 **Supplementary Table 1.** Overview of available studies on the comprehensibility of gender-fair language.

**source**	**exp.**	**sample**	***N***	***k***	**experimental conditions / language forms**	**text's topic**	**text length in words**	**gendered phrases**
Blake & Klimmt (2010)	1	university students	201	3	masculine-only forms,<br>mix of internal-I-forms and neutral forms,<br>mix of pair forms and neutral forms	newspaper article on chlorine gas in the gym	349	15
Blake & Klimmt (2010)	2	mostly university students	325	2	masculine-only forms,<br>mix of internal-I-forms and neutral forms	newspaper article on demonstrations for better working conditions	340	15
Braun et al. (2007)	-	Broadly diversified educational back-ground, 17-62 years	86	3	masculine-only forms,<br>internal-I-forms,<br>mix of pair forms and neutral forms	package insert medication	170	6
Frank-Cyrus & Dietrich (1997) *	-	mostlyy 40-63 years, 84% with at least a high school diploma	734	3	masculine-only forms,<br>pair forms,<br>neutral forms	long-term care insurance law	163	15
Friedrich & Heise (2019)	-	mostly university students	356	2	masculine-only forms,<br>pair forms	electricity supply contract	938	39
Friedrich et al. (2021)	1	mostly university students	159	2	masculine-only forms,<br>gender star in its plural form	board game instructions	462	20
Friedrich et al. (2021)	2	mostly university students	127	2	masculine-only forms,<br>gender star in its singular form	kabbadi sport	471	48
Friedrich et al. (2024)	1	mostly university students	133	3	masculine-only forms,<br>gender star in its plural form,<br>gender star in its singular form	first aid	749	67
Friedrich et al. (2024)	2	mostly university students	110	3	masculine-only forms,<br>gender star in its plural form,<br>gender star in its singular form	kabbadi sport	458	51

Friedrich et al. (2022)	-	mostly university students	97	2	masculine-only forms, glottal stop	self-determination theory	1056	27
Gygax & Gesto (2007)	-	university students	40	4	masculine-only forms, neutral forms, pair forms, slash-forms	job descriptions	743	15
Jöckel et al. (2021)	1	all age groups, level of education not specified	770	4	masculine-only forms, pair forms, neutral forms, glottal stop	news program	153	9
Jöckel et al. (2021)	2	children between 8-14 years old, *Md* = 10	137	4	masculine-only forms, pair forms, neutral forms, glottal stop	news program	not stated	not stated
Klimmt et al. (2008)	1	university students	41	2	masculine-only forms, slash-forms	newspaper article on medical strike	445	23
Klimmt et al. (2008)	2	university students	67	2	masculine-only forms, slash-forms	newspaper article on medical strike	not stated	not stated
Pabst & Kollmayer (2023)	-	academics & non-academics	163	2	masculine-only forms, gender star	travel tips Mallorca	234	10
Pöschko & Prieler (2018)	-	vocational students	137	3	masculine-only forms, slash-forms, neutral forms	textbook text on project management	not stated	not stated
Rothmund & Christmann (2002)	-	all levels of education, but mostly higher educational backgrounds	220	4	masculine-only forms, masculine-only forms plus footnote, mix of masculine-only forms und pair forms, mix of masculine-only forms and neutral forms	thermal baths in Budapest	900	28
Steiger-Loerbroks & von Stockhausen (2014)	-	mostly university students	45	2	masculine-only forms, neutralforms	legal texts from the Civil Code	not stated	not stated

Note. If several experiments were reported in the corresponding source, the column “exp.” indicates which experiment is reported in this row. The column “*k*” indicates the number of experimental conditions of the respective study. * In this case, the study is not an experiment.

Supplementary Table 2. Results of the ANOVAs.

**Factor**	**Language form**					**gender**					**interaction**				
**dependent variable**	$df_{between}$	df_{within}	F	**partial** η^2	p	$df_{between}$	df_{within}	F	**partial** η^2	p	$df_{between}$	df_{within}	F	**partial** η^2	p
subjective compre-hensibility	1	262	1.03	.00	.31	1	262	6.27	.02*	.01	1	262	0.00	.00	.97
word difficulty	1	261	0.15	.00	.70	1	261	0.07	.00	.80	1	261	0.70	.00	0.40
sentence difficulty	1	261	0.95	.00	.33	1	261	0.49	.00	.49	1	261	0.17	.00	.68
aesthetic appeal	1	262	1.20	.00	.27	1	262	6.18	.02*	.01	1	262	0.05	.00	.83

Note. * $p < .05$

3 **Supplementary Table 3.** Comments by the participants on the video or the study.

Comment by the participants	Translation
(anonomized) hast du richtig toll gemacht!	(anonomized) you've done a really great job!
1. Sehr interessantes Thema. da dieses Thema in den Medien stark polarisiert 2. Vielleicht sollte die eigene Sprachkompetenz des Zuschauers eingebunden werden (Bsp. Ausländer mit A1 bis C2 Schein. oder jemand mit Sprach oder Lesebehinderung) 3. Meine	1. very interesting topic. as this topic is highly polarized in the media 2. perhaps the viewer's own language skills should be included (e.g. foreigners with A1 to C2 certificate. or someone with a speech or reading disability) 3. My
Das Video war etwas leise. obwohl auf voller Lautstärke abgespielt. Als Lernvideo ist es etwas sehr textlastig - Das Bild am Anfang als Animation (Einblendung der einzelnen Schichten. passend zu den sprachlichen Erläuterungen) fände ich gut.	The video was a bit quiet, although it was played at full volume. As an educational video, it is a bit text-heavy - I would like to see the image at the beginning as an animation (fading in the individual layers to match the verbal explanations).
Das Video war für mich persönlich wirklich kaum zu ertragen. Das schlimmste ist es wenn aufgrund des genderns der Sinn eines Satzes mehrdeutig wird. oder vollkommen verloren geht. Wenn Sie beispielsweise von Athlet:innenen im Plural reden. was überhau	For me personally, the video was really hard to bear. The worst thing is when the meaning of a sentence becomes ambiguous or completely lost due to the gendering. For example, when you talk about athletes in the plural.
Dem Video zu folgen war sehr anstrengend aufgrund der monotonen und emotionslosen Sprechweise.	Following the video was very tiring due to the monotonous and emotionless way of speaking.
Der Ton im Video war leider zwischendurch etwas schlecht und die Folie mit praktischen Empfehlungen war viel zu kurz eingeblendet. es blieb keine Zeit die Inhalte zu lesen. Ansonsten war das Video imformativ und lehhreich. Hat ein paar gute Tipps. :)	Unfortunately, the sound in the video was a bit poor at times and the slide with practical recommendations was far too short. there was no time to read the content. Otherwise, the video was informative and instructive. Has some good tips. :)
Der Ton im Video war sehr leise verglichen mit der durchschnittlichen Lautstärke in Videos auf z.B. YouTube.	The sound in the video was very quiet compared to the average volume in videos on YouTube, for example. There was also a slight

Außerdem war die ganze Zeit über ein leises Knacken und Knistern zu hören. Vielleicht lag es auch nur am Browser. das kann ich nicht überprüfen	cracking and crackling sound the whole time. Maybe it was just the browser. I can't check that
Die Audio Qualität war nicht angenehm. Hätte fast aufgehört das Video zu schauen. Viel Erfolg für die weitere Arbeit!	The audio quality was not pleasant. I almost stopped watching the video. Good luck with your future work!
Die Folie mit den Praxistipp wurde sehr schnell durch geklickt. Der Restliche Vortrag war gut	The slide with the practical tip was clicked through very quickly. The rest of the presentation was good
Die Folie vor der Literatur war mir zu kurz dargestellt	The slide before the literature was too short for me
Die Folien im Video haben zu viel Text. man ist verleitet diesen zu lesen und wird so von Vortrag abgelenkt.	The slides in the video have too much text. you are tempted to read it and are thus distracted from the lecture.
Die Präsentation war super langweilig. aber gut aufgebaut. Es war eindeutig zu viel Text in den Folien. Alles in einem könnte man es kürzer fassen. viel Spaß bei deiner Studie	The presentation was super boring. but well structured. There was definitely too much text in the slides. All in all, it could be shorter. have fun with your study
Die Präsentation hat eine Seite/Folie 5. die kurz gezeigt. aber nicht weiter erklärt wurde.	The presentation has one page/slide 5. which was shown briefly. but not explained further.
Die Tonqualität gepaart mit der monotonen Vortragsart sind jedenfalls nicht unbedingt hilfreich zum lernen. Zusätzlich viel zu leise. Ich wollte nach 1 Minute eigentlich abschalten. Wenn das Tempo zwischendurch springt. weil Inhalte nur durchgeklickt	The sound quality paired with the monotonous presentation style are not necessarily helpful for learning. In addition, much too quiet. I actually wanted to switch off after 1 minute. If the tempo jumps in between. because content just clicked through
Die Tonqualität könnte stellenweise besser sein.	The sound quality could be better in places.
Eine gute Umfrage. welche zeigt. wie sehr die Aufmerksamkeit bei schlechter Lernstoffpräsentation in den Keller geht. Die	A good survey. which shows how much attention goes down when learning material is poorly presented. Attention spans have

Aufmerksamkeitsspanne ist über die Jahre durch Online-Medien extrem zurückgegangen. Ich persönlich leide auch darunter. da ich m	decreased dramatically over the years due to online media. I personally suffer from this as well.
Eingangs wurde die Gliederung zwei Mal sprachlich wiederholt und die Tipps am Ende sind leider viel zu schnell angezeigt worden.	The outline was repeated twice at the beginning and the tips at the end were unfortunately displayed far too quickly.
ergänzende Visualisierung in PowerPoint einbauen	Incorporate additional visualization in PowerPoint
Es ist irritierend. dass einmal eine Grafik und am Ende die Tipps ganz kurz eingeblendet werden. ohne dass dazu gesagt wird. dass dazu kein Kommentar kommen wird. sondern dass man sich das bei Interesse selbst angucken soll. So wartet man und dann ist di	It is irritating that a graphic is shown once and the tips are shown very briefly at the end. without saying that there will be no commentary, but that you should look at it yourself if you are interested. So you wait and then the
Es war sehr viel Text im Viedeo. Ich habe nur zu gehört ohne auf das Viedeo groß zu Achten.	There was a lot of text in the video. I just listened without paying much attention to the video.
Gute Zusammenfassung zum Thema Lernen . Die praktischen Tipps haben mich sehr an mein eigenes Studium erinnert!	Good summary on the subject of learning. The practical tips reminded me a lot of my own studies!
Habe etwas dazugelernt Z.B. -dass mehrfaches Lesen nicht optimal ist -Lernen über einen längeren Zeitraum effektiver und nachhaltiger ist als geballtes Pauken	I learned something new, e.g. -that repeated reading is not optimal -learning over a longer period of time is more effective and sustainable than concentrated cramming
Hallo. bei der Wiedergaber knistert es ganz leicht. Das ist störend und sollte mit einem besseren Aufnahmegerät behoben werden können.	Hello, there is a slight crackling noise when you play back. This is annoying and should be remedied with a better recording device.
Hallo. es wird zu viel Text vorgelesen. lesen kann jeder selbst. Ich würde mehr Grafikelemente einbauen. So wird eine weitere Ebene	Hello. too much text is read out loud. everyone can read for themselves. I would incorporate more graphic elements. This would

angesprochen um die Inhalte zu vertiefen. Also Sprache. lesen (selbst lesen) und Bilder.	address another level to deepen the content. So language. reading (read it yourself) and pictures.
Hallo. mir ist aufgefallen. dass der Lernende verwendet wurde. Die männlichen Form. es wäre schön gewesen auch die Weibliche zu lesen. Vielen Dank und viele Grüße	Hello. I noticed that the learner was used. The masculine form. it would have been nice to read the feminine as well. Many thanks and best regards
Ich finde es braucht mehr Bilder und Stichworte anstatt ganze Sätze	I think it needs more pictures and keywords instead of whole sentences
Ich finde Ihre Idee und das Video sehr gut und verständlich. Gute erklärstimme für Videos. Viel Erfolg bei der Studie.	I think your idea and the video are very good and easy to understand. Good explanatory voice for videos. Good luck with the study.
Ich habe etwas anderes nebenbei gemacht. Dadurch war es nicht schlimm das. dass Video (wohl absichtlich) langweilig war. Die Corona Lernerfahrung machts möglich.	I was doing something else on the side. So it was not bad that the video was (probably intentionally) boring. The Corona learning experience makes it possible.
Ich hätte mir mehr Fragen zur Konzentrationsfähigkeit gewünscht. Vor allem. wenn man am Handy ist und z.B. durch Benachrichtigungen abgelenkt wird. Allgemein Fragen zum Inhalt erwartet (aber hier lag das Interesse woanders?) Zweitens. das Video kon	I would have liked more questions on the ability to concentrate. Especially when you are on your cell phone and are distracted by notifications, for example. Generally expected questions about the content (but here the interest was elsewhere?) Secondly, the video con
Ich konnte die Frage zu Was glauben sie wurde mit der Befragung untersucht nicht beenden. weil ich aus versehen auf weiter geklickt habe. Ich bin der Meinung. dass die Sprache und die Art der visuellen Darstellung im Vergleich zum Erzählen untersuc	I couldn't finish the question on What do you think they were investigating with the survey. because I accidentally clicked continue. I am of the opinion that the language and the type of visual representation compared to the narrative was examined.
Ich weiß nicht. ob das so sein sollte. aber es gab einige Rechtschreibfehler in der Präsentation und einige Folien wurden	I don't know if it should be. but there were some spelling mistakes in the presentation and some slides were skipped. Some terms were

übersprungen. Einige Begriffe wurden gegendert. einige nicht - sowohl schriftlich als auch verbal. Das war sehr verwirrend. wenn	gendered. some were not - both written and verbal. It was very confusing. when
Ich weiss nicht. ob es Absicht war. aber bei dem Video hat man immer ein Rauschen oder Kratzen während des Gesprochenen (Tonspur) gehört. Das war recht störend und anstrengend der lieben Frau (anonomized) zuzuhören. Das habe ich bei der Beantwortung jed	I don't know if it was intentional, but in the video you always heard a hissing or scratching during the speech (soundtrack). That was quite annoying and exhausting to listen to the dear woman (anonomized). I noticed that when answering each
Im Video sind einzelne Folien viel zu kurz dargestellt.	In the video, individual slides are much too short.
Im Video waren Fehler enthalten. Manchmal wurden Aussagen doppelt getätigt oder das einblenden von Inhalten war zu früh und passte nicht zu dem Gesagten. Des Weiteren war es sehr. sehr öde gestaltet sowie zum Ende wurde das was eigentlich Interessant	There were mistakes in the video. Sometimes statements were made twice or the fading in of content was too early and did not match what was said. Furthermore, it was very dull and at the end, what was actually interesting was not shown.
In dem Video fehlt bei zwei Folien der Ton und bei einer werden die Stichpunkte nicht nacheinander eingeblendet. Ich weis nicht. ob das Absicht ist.	In the video, the sound is missing in two slides and in one the key points are not shown one after the other. I don't know if this is intentional.
In Zukunft die Folien vielleicht nicht nur ablesen. Das habe ich schon in der 5. Klasse gelernt.	Maybe don't just read the slides in future. I already learned that in 5th grade.
Interessant	Interesting
Kleiner Tipp: Wenn schon gegendert wird. dann bitte a) dauerhaft und b) korrekt - beide genannten Geschlechter müssen in den Satzbau passen. Beispiel: Die Student:innen lernen auf unterschiedliche Art und Weise wäre nicht korrekt. da der männli	A little tip: If you're going to use gendered language, then please make it a) permanent and b) correct - both genders must fit into the sentence structure. Example: The students learn in different ways would not be correct.

Kritik: • Langweiliger PowerPoint • Eintönige Stimme • Welche Zielgruppe? • schlechte Mikrofon • Anstrengend zuzuhören • Zu viel Text Ab Folie zwei wurde es dann inhaltlich interessant. Aber geht man davon aus. dieses Video auf YouT	Criticism: - Boring PowerPoint - Monotonous voice - Which target group? - Poor microphone - Tiresome to listen to - Too much text From slide two onwards, the content became interesting. But assuming this video on YouT
Leider wurden im Video einmal eine Folie und die Informationen zu Teil 4. Praktische Empfehlungen zu schnell übersprungen. Man kann zwar auf Stop drücken. ist aber nicht komfortabel.	Unfortunately, a slide and the information on part 4 Practical recommendations were skipped too quickly in the video. You can press stop, but it is not convenient.
Manches war schwer verständlich. weil Video und Gesprochenes nicht zu einander passten. Manche Texte verschwanden zu schnell. Die Stimme habe Ich manchmal als zu leise empfunden.	Some things were difficult to understand because the video and spoken words did not match. Some texts disappeared too quickly. I sometimes found the voice too quiet.
Mehr Kontraste in der Präsentation verwenden.	Use more contrast in the presentation.
Mich hat es ziemlich irritiert. dass die Lautstärke der Stimme ziemlich variiert. Hätte man in der Nachbearbeitung etwas darauf achten sollen.	I found it quite irritating that the volume of the voice varied quite a bit. Should have paid more attention to this in post-production.
Mir ist aufgefallen. dass gar nicht gegendert wurde! Teilweise hat mich das sogar beim Zuhören verwirrt und durcheinander gebracht. Viel Spaß beim Ergebnisse auswerten! :-)	I noticed that it wasn't gendered at all! Sometimes it even confused me while listening. Have fun evaluating the results! :-)
Mir ist gendergerechte Sprache wichtig. Allerdings empfinde ich sie wie z.B. im Lehrvideo störend. Sinnvoll ist für mich daher eine geschlechtsneutrale Variante wie z.B Studierende. die grammatikalisch nicht fehlerhaft ist.	Gender-appropriate language is important to me. However, I find it distracting, as in the instructional video, for example. It therefore makes sense to me to use a gender-neutral version such as Studierende. that is not grammatically incorrect.
nein	No

nein	No
Nö	No
Plz mehr gendern ansonsten war's nice	Plz more gendern otherwise it was nice
Rechtschreibfehler bei dem ersten oder zweiten Kapitel am unteren Ende der Seite. da wurde von Textmarkierungen von Dozenten/Lehrenden geredet. Der Rechtschreibfehler war im Wort vorgenommene .	Spelling error in the first or second chapter at the bottom of the page. there was talk of text marking by lecturers/teachers. The spelling mistake was made in the word .
Sehr nice gemacht (anonomized) ♡ LG (anonomized)	Very nicely done (anonomized) ♡ LG (anonomized)
Sehr schöner optischer Aufbau der Präsentation!	Very nice visual structure of the presentation!
Sogleich mir die Inhalte nicht unbekannt waren. fand ich es schön. die Tipps zum Lernen mal wieder aufzufrischen. Tolles Thema!	Although I was not unfamiliar with the content, I found it nice to refresh the tips for learning. Great topic!
Statt Text würde ich mehr Bilder mit rein nehmen. das macht das ganze anschaulicher und nicht ganz so Text lastig. Oder eher Stichworte schreiben. statt ganze Sätze.	Instead of text, I would include more pictures. that makes the whole thing more vivid and not quite so text-heavy. Or write keywords instead of whole sentences.
Studierende anstatt Studenten schreiben oder auf der Präsentation einen entsprechenden Vermerk anbringen. „Danke für eure Aufmerksamkeit" Folie am löschen. Nach einem Lehrvortag sollte man so etwas vermeiden :) Ggf. weniger Text auf den Folie	Write students instead of students or make a corresponding note on the presentation. "Thank you for your attention" slide at the end. You should avoid this after a lecture :) Less text on the slide if necessary
Verschiedene Mikrofonaufnahmequalitäten- geschlechter gerechte Sprache lästig. oft klang es auch eher nur nach den Frauen(Pause	Different microphone recording qualities - gender-equitable language annoying. often it sounded more like women only (pause

fehlte)- generisches Maskulinum ist Teil der grammatikalisch richtigen Sprache. also nutzen wir es :-)	was missing) - generic masculine is part of grammatically correct language. so let's use it :-)
viel erfolg	Good luck
Viel Erfolg bei der Bachelor-Arbeit! :)	Good luck with your bachelor thesis! :)
Viel erfolg!	Good luck!
Visualisierung des Themas durch ein sogenanntes Storyboard hätte die ansonsten sehr trocken dargestellte Theorie lebhafter und nachvollziehbarer gestaltet. Man hätte dabei beispielsweise eine direkte Zuhörer:in Ansprache wählen können und direkt mit	Visualizing the topic with a so-called storyboard would have made the otherwise very dry theory more lively and comprehensible. For example, you could have chosen a direct address to the listener and directly talked to them.
Vor den Fragen mit der geschlechterspezifischen Sprache wurde nicht deutlich ob es sich nun um Sprache handelt. welche lediglich das biologische Geschlecht (Männlich. Weiblich) mit einschließt handelt. oder ob mit den Fragen ein bezug zu den neusten En	Before the questions with the gender-specific language, it was not clear whether this was language that only included the biological gender (male, female) or whether the questions were related to the latest developments in the field.
Wäre es nicht schlau gewesen wenn Leute bei der Frage. ob sie das Video angesehen haben mit Nein antworten. die Umfrage für diese Personengruppe zu beenden?	Wouldn't it have been clever if people had answered no to the question of whether they had watched the video. to end the survey for this group of people?
Wichtiges Thema! Gute Frage Arbeit!	Important topic! Good question Work!
Zu der Präsentation an sich: Ich habe ja ausgewählt. dass ich teilweise nicht unbedingt die Fachwörter verstanden habe (besonders bei der Definition). Meines Erachtens könnte es da helfen. komplexe Sätze in einem Nebensatz kurz in eigene Worte	Regarding the presentation itself: I selected that I didn't necessarily understand some of the technical terms (especially in the definition). In my opinion, it could help to briefly translate complex sentences into your own words in a subordinate clause.
